# Welcome to *Fungal Biology and Biotechnology*

**DOI:** 10.1186/s40694-014-0008-5

**Published:** 2014-10-14

**Authors:** Alexander Idnurm, Vera Meyer

**Affiliations:** 1 School of Botany, University of Melbourne, Melbourne, 3010 VIC Australia; 2 Institute of Biotechnology, Berlin University of Technology, Berlin, 13355 Germany

## Abstract

No abstract

Fungi are fundamental and predominant influences in our lives. These species impact us in diverse ways, such as generating the food we eat and drink, providing life-saving pharmaceutical agents, and are sources of enzymes; and yet they adversely affect the structural integrity of our buildings, poison us, cause common mycoses and in not so rare cases can kill us, and they are the principal group of microbes responsible for plant diseases that threaten global food security [1],[2]. While fungi are described as “microbes”, this term may conjure images of a bacterium like *Escherichia coli* growing as a colony in a petri dish culture or as a microscopic rod. Some fungi do grow in an equivalent manner, yet many others have intricate developmental forms and in relative size some are best appreciated at the scale achieved from an airplane rather than at electron microscope resolution [3],[4].

Not surprisingly given their importance to humans, some species of fungi have been studied extensively. For instance, the yeast *Saccharomyces cerevisiae* and its close relatives are used in baking and brewing: *S. cerevisiae* was the first eukaryote with a genome sequence and became the premier model species to understand large parts of eukaryotic molecular biology. However, the realm of fungal biology is far vaster and almost entirely unexplored beyond a handful of model species, with some estimates of up to 5 million fungal species on the planet [5]. The reduction in DNA sequencing costs and the implementation of functional studies through gene manipulations are allowing hitherto unexplored fungi to reveal insights into their biology, at a level that would have been barely dreamed of for the model species less than a decade ago. This is a new golden age of discovery in the fungi, and an exciting time to take part of this adventure and then describe these discoveries for future generations.

While our appreciation of fungi grows daily, the communication of this new found knowledge has become scattered across the publishing world. The journals dedicated to mycology tend to focus on specific aspects of their biology. Journals dedicated to fungal biotechnology do not exist. Furthermore, major changes have occurred in the last decade in how journals are organized and in requirements of funding agencies to make data accessible to the general public funding the research. Data have changed from being presented in print form to cases being best visualized online as large datasets, videos, or three-dimensional images. The availability of internet resources has shifted philosophy to include and rely more on online supplemental material. Another trend is the way in which information is sought and identified – gone is the casual browsing of latest issues in a library! – and providing interconnectivity between research areas is increasingly important [6]. One impact of these changes on publishing has been the rise of online and open-access journals [7],[8]. Some of these journals were established without the necessary commitment to ensure that thorough science appears on their pages [9], and hence there is a desire for any new journals to be founded by reputable publishing organizations.

Within the context described above, *Fungal Biology and Biotechnology* bridges a need for a venue that is inclusive of all aspects of the fungi and the application of fungi or their products to our lives. It is produced by BioMed Central, a publisher with more than a decade of experience in ensuring the rigor of the peer-review process and quality in production of the final papers. The journal has an editorial board of internationally-recognized experts in all areas of fundamental and applied fungal research [10], acting to ensure thorough peer-review of the articles. The mission of the journal is to bring high quality insights into fundamental aspects of the biology of the fungi and the practical applications of that information met through biotechnological innovations. We intend for the journal to become a hub for researchers seeking information on their favorite topics as well as those considering new or alternative directions. The journal shall become a platform for scientists from academia and industry to present their hottest findings in unicellular or multicellular fungal systems, in medical or industrial strains, and in so far unexplored species. This will be a platform for experts to discuss their visions on how fungi can help us to address some of the key challenges of the 21^st^ century: how the innovation and technology gap in antibiotics research can be bridged, how humankind can live with in limits, i.e. how commodities and high value products can be produced from renewable resources, including waste, to move to a post-petroleum future.

The journal’s first article is written by one of the legends of fungal biology, Claudio Scazzocchio. Claudio traces and comments on the developments in biology since he first started working with fungi in the 1960s [11]. Claudio’s career has many remarkable features, with one illustration being the ability to publish on the same topic (in this case purine assimilation in *Aspergillus*) for 50 years. His experiences provide perspective to outline the remarkable changes that occurred during this time, and how the current genomics revolution is influencing biology as evident by its effects on fungal biology. As editors, we already see the legacy of the changes in biological research that Claudio has highlighted within the first research articles submitted to the journal. We look forward to bringing you this and future research, and encourage you to share your next insights about the fungi in *Fungal Biology and Biotechnology*.
